# Comparison of the effectiveness of ultrasound-guided radiofrequency ablation and conventional open thyroidectomy in the treatment of benign thyroid nodules

**DOI:** 10.12669/pjms.40.7.9486

**Published:** 2024-08

**Authors:** Yuehe Fu, Yuke Xia, Haiyan Wang, Gong Zhang

**Affiliations:** 1 Yuehe Fu Department of Thyroid and Breast Surgery, The Affiliated Jiangning Hospital of Nanjing Medical University, 169 Hushan Road, Nanjing, Jiangsu Province 211100, P.R. China; 2 Yuke Xia Department of Thyroid and Breast Surgery, The Affiliated Jiangning Hospital of Nanjing Medical University, 169 Hushan Road, Nanjing, Jiangsu Province 211100, P.R. China; 3 Haiyan Wang Department of Thyroid and Breast Surgery, The Affiliated Jiangning Hospital of Nanjing Medical University, 169 Hushan Road, Nanjing, Jiangsu Province 211100, P.R. China; 4 Gong Zhang Department of Thyroid and Breast Surgery, The Affiliated Jiangning Hospital of Nanjing Medical University, 169 Hushan Road, Nanjing, Jiangsu Province 211100, P.R. China

**Keywords:** Ultrasound-guided, Radiofrequency ablation, Conventional open thyroidectomy, benign thyroid nodules

## Abstract

**Objective:**

To compare the effectiveness of ultrasound (US)-guided radiofrequency ablation (RFA) and conventional open thyroidectomy (OT) in the treatment of benign thyroid nodules (BTN).

**Methods:**

Medical records of 103 patients with BTN undergoing surgical treatment at The Affiliated Jiangning Hospital of Nanjing Medical University from March 2019 to March 2022 were retrospectively analyzed. Records show that 53 patients underwent US-guided RFA (observation group) and 50 patients underwent conventional OT (control group). Perioperative indicators (operation duration, intraoperative blood loss, postoperative hospital stay, incision length, and VAS score 12h and 24h after surgery), complications, thyroid function, and nodule recurrence in both groups were compared and analyzed.

**Results:**

Perioperative indicators of patients in the observation group were better, and the visual analogue scale (VAS) scores at 12 and 24 hours after the surgery were lower than those of the control group (*p*<0.05). The incidence of complications in the observation group was significantly lower than that in the control group (*p*<0.05). There was no statistically significant difference in the preoperative levels of thyroid-stimulating hormone (TSH), serum free thyroxine (FT4) and serum free triiodothyronine (FT3) between the two groups (*p*>0.05). The postoperative TSH levels in the observation group increased compared to the preoperative levels and were higher than those in the control group, while FT4 and FT3 levels decreased after surgery and were lower than those in the control group (*p*<0.05).

**Conclusions:**

Compared to conventional open thyroidectomy, US-guided RFA is associated with less trauma, faster recovery, fewer complications, and less impact on thyroid function in the treatment of patients with BTN.

## INTRODUCTION

Benign thyroid nodules usually develop as a result of overgrowth of normal thyroid tissue, which accounts for over 90% of all identified thyroid nodules.^1^ Although the prognosis of benign thyroid nodule (BTN) is good, they may lead to compression symptoms, such as voice changes, difficulty swallowing, and local protrusions may affect neck aesthetics.^1^,^2^ Moreover, studies have shown that nearly 20% of BTN have a risk of subsequent malignancy.^3^

Surgery is a common treatment method for BTN. Open thyroidectomy (OT) is a routine procedure that involves removing nodules in an open state.^3^,^4^ Although the therapeutic effect is reliable, the surgical trauma of OT is significant and may result in large scars.^4^,^5^ Radiofrequency ablation (RFA) has emerged as a commonly used minimally invasive treatment method for BTN in recent years.^5^,^6^ The thermal energy, associated with radiofrequency ablation, causes localized protein coagulation, tumor cell necrosis, and gradual lesion absorption. RFA has the advantage of minimal trauma and fast recovery.^6^,^7^

In recent years, ultrasound (US)-guided RFA has been widely used in the treatment of BTN. A large number of clinical studies have shown that US-guided RFA is associated with over 80% postoperative nodule reduction rate after one year of follow-up, and over 90% postoperative nodule reduction rate after two years of follow-up.^8^,^9^ However, there is currently a lack of clinical research comparing US-guided RFA with conventional OT in the treatment of BTN. This study aimed to compare and analyze the effects of US-guided RFA and conventional OT in the treatment of patients with BTN to provide reference suggestions for clinicians.

## METHODS

Clinical records of 103 patients who underwent BTN resection at The Affiliated Jiangning Hospital of Nanjing Medical University from March 2019 to March 2022 were retrospectively analyzed. Among them, 53 patients underwent US-guided RFA were assigned to the observation group, and 50 patients underwent conventional OT were assigned to the control group.

### Ethical Approval:

The Medical Ethics Committee of our hospital approved this study (No. KY-2022-023, Date: Aug. 25^th^ 2023).

### Inclusion criteria:

- Met the diagnostic criteria for BTN, and BTN should be confirmed as benign by two separated fine-needle aspiration or core-needle biopsy.^10^
- Normal thyroid function indicators.
- Complete medical record and follow-up information.

### Exclusion criteria:

- Malignant thyroid nodules.
- Bilateral thyroid nodules.
- Severe underlying diseases, combined with organ dysfunction.
- Presence of other throat diseases.
- Patients with a history of thyroid surgery.
- Patients with coagulopathy.
- The nodule is located behind the sternum.

### Ultrasound-guided radiofrequency ablation:

The instruments used were PHILIPS iU22 ultrasound system with probe L12-5 (Philips Medical Systems, Bothell, WA, USA) and multipole RFA instrument (LDRF-120S, Mianyang Lide Electronics Co., Ltd., Mianyang, China). Ablation was performed by an interventional consultant radiologist with six years’ experience. After completing appropriate preoperative examinations, patient’s posture was adjusted to supine position, the head was extended backward, fully exposing the neck. Neck infiltration anesthesia was initiated after routine disinfection and towel laying. Under ultrasound guidance, electrodes were inserted into the nodules and RFA was performed. During the treatment process, an appropriate amount of 0.9% sodium chloride was injected into the thyroid capsule to form a “liquid isolation zone”. If the nodule was close to important tissues such as the laryngeal nerve, esophagus, etc., special attention was paid to protect the adjacent tissue. Complete ablation was not recommended. If the nodule contained cystic components or fluid, it was fully aspirated.

The ablation zone was determined according to the nodule condition shown by US examination. The power was maintained at 30~120W for 3~5s each time, and the ablation was generally completed within 40 minutes. After satisfactory ablation effect was achieved, absence of bleeding was confirmed, by the US, and desmopressin acetate was injected to stop the bleeding. The puncture site was treated, local compression was applied to stop the bleeding, and cold was applied to the skin for 30 minutes.

### Conventional open thyroidectomy:

OT was performed for all patients by the same surgical team. Patients were instructed to lie on their back. Pillows were placed on their shoulders to extend the neck and fully expose the surgical area. After routine disinfection and tissue laying treatment, an arc incision was made about 2cm above the sternum, and skin and subcutaneous tissues were dissected layer by layer until the thyroid was fully exposed along the midline of the neck. Based on the specific situation of the nodules, detected during the surgery, the mass was dissociated, completely removed, and bleeding stopped. A drainage tube was inserted, the incision sutured, and the surgery was completed.

### Observation indicators:

- Perioperative indicators, including operation duration, intraoperative bleeding loss, postoperative hospital stay, incision length, and postoperative pain at 12 and 24 hours. Pain was evaluated using visual analogue scale (VAS). Scores are recorded by making a handwritten mark on a 10-cm line that represents a continuum between “no pain” and “worst pain.”^11^
- The occurrence of surgical complications, including hematoma formation, hoarse voice, parathyroid injury, infection.
- TSH, TT4, and FT3 levels before and six months after the surgery. UniCel Dxl800 automatic electrochemiluminescence immunoassay (Beckman Coulter, USA) and matching reagents were used for detection.
- Recurrence of nodules 12 months after the surgery.

### Statistical analysis:

Data were put into Microsoft Excel and analyzed using SPSS version 26.0 (IBM Corp, Armonk, NY, USA). The normality of the data was evaluated using the Shapiro Wilk test. The data of normal distribution were expressed as mean ± standard deviation. Independent sample *t*-test was used for intergroup comparison, and paired *t*-test was used for intragroup comparison before and after the surgery. The data of non-normal distribution were expressed by median and interquartile interval, and Mann Whitney *U* test was used for intergroup comparison. The counting data were represented by the number of use cases, and Chi-squared test was used. *p*<0.05 suggested that the difference is statistically significant.

## RESULTS

A total of 103 patients were included in this study, including 61 males and 42 females. Age of the patients ranged from 32 to 68 years old, with an average of 46.8 ± 8.4 years. Patients (n=53) who underwent US-guided RFA comprised the observation group, and 50 patients who underwent traditional OT comprised the control group. There was no statistically significant difference in the baseline data between the two groups (*p*>0.05) (Table-I). The observation group had shorter operation duration and hospital stay compared to the control group, less intraoperative bleeding, shorter incision length, and lower VAS scores at 12 and 24 hours postoperatively (*p*<0.05) (Table-II).

**Table-I:** Comparison of general data between the two groups.

Group	Gender (Male/Female)	Age (years)	Nodule volume(cm^3^)	Nodule properties (n)		

				Solid nodules	Cystic nodules	Mixed nodules
Control group (n=50)	26/24	46.2±7.8	3.51±0.69	26	17	7
Observation group (n=53)	35/18	47.4±8.9	3.65±0.80	18	26	9
*χ^2^*/*t*	2.099	-0.726	-0.919	3.504		
p-value	0.147	0.470	0.360	0.173		

**Table-II:** Comparison of operation conditions between the two groups.s

Group	Operation duration (min)	Intraoperative blood loss (ml)	Postoperative hospital stay (d)	Incision length (mm)	VAS score 12h after surgery	VAS score 24h after surgery
Control group (n=50)	49.6±10.6	17.4±2.5	4.80±1.14	28.7±2.9	6(5, 7)	4(3, 5)
Observation group(n=53)	25.8±4.3	4.37±1.33	1.51±0.41	2.65±0.40	3(3, 4)	2(2, 3)
t/Z	14.753	33.058	19.223	62.414	-7.498	-5.420
p-value	<0.001	<0.001	<0.001	<0.001	<0.001	<0.001

The incidence of complications in the observation group was 1.9%, lower than the 12.0% in the control group (*p*<0.05) (Table-III). Before the surgery, there was no statistically significant difference in the levels of TSH, TT4, and FT3 between the two groups (*p*>0.05). Six months after the surgery, preoperative and postoperative levels of TSH, TT4, and FT3 were comparable in the observation group (*p*>0.05); However, the postoperative TSH level in the control group significantly increased compared to before the surgery and was higher than that in the observation group. Postoperative levels of TT4 and FT3 were lower than before the surgery and lower than those in the observation group (*p*<0.05) (Table-IV). Follow-up to 12 months after surgery showed no recurrence of nodules in both groups.

**Table-III:** Comparison of complication rate between the two groups.

Group	Complication				Total

	Hematoma formation	Hoarseness	Parathyroid gland injury	Infection	
Control group (n=50)	1 (2.0)	2 (4.0)	2 (4.0)	1 (2.0)	6 (12.0)
Observation group(n=53)	0 (0.0)	1 (1.9)	0 (0.0)	0 (0.0)	1 (1.9)
*χ^2^*	-	-	-	-	4.154
*p-value*	-	-	-	-	0.042

**Table-IV:** Comparison of thyroid function between the two groups.

Group	TSH (mU/L)		FT4 (pmol/L)		FT3 (pmol/L)	

	Preoperative	6-months after surgery	Preoperative	6-months after surgery	Preoperative	6-months after surgery
Control group (n=50)	7.22±0.69	8.05±1.08*	18.55±1.98	16.31±1.83*	6.77±0.73	6.11±0.72*
Observation group(n=53)	7.16±0.73	7.24±1.02	18.83±2.23	18.67±2.10	6.62±0.67	6.53±0.67
*t*	0.411	3.922	-0.659	-6.064	1.067	-3.036
*p-value*	0.682	<0.001	0.512	<0.001	0.289	0.003

***Note:*** Compared with preoperative data in this group,

* p<0.05.

## DISCUSSION

The results of this study show that compared with conventional open thyroidectomy, US-guided RFA has advantages of minimal trauma, faster recovery, fewer complications, and lower impact on thyroid function in patients with BTN, which is basically consistent with the findings by Yan et al.^12^ Elhefny et al^13^ also found that pain was more acceptable in RFA than surgery with few complications.

Numerous reports show that US-guided RFA approach in treating BTN is associated with less trauma, fewer complications, and can retain thyroid function.^9^,^14^ The study by Jeong *et al*.^15^ found that patients receiving RFA had shorter operation duration, shorter postoperative hospital stay, smaller incisions, less intraoperative bleeding, lower postoperative pain, and lower incidence of complications, which is consistent with our results. It is suggested that RFA has advantages such as minimal trauma, fast recovery, and fewer complications in the treatment of BTN. US-guided RFA is performed under real-time ultrasound guidance, and requires only puncture, without the need for a neck incision. Therefore, it can effectively shorten the surgical. In contrast, OT requires large incision for the removal of thyroid lobes, parathyroid glands, etc., and causes significant trauma with greater amount of intraoperative bleeding, prolonged postoperative recovery time, and increased risk of complications.^16^,^17^ Fung *et al*.^18^ conducted long-term follow-up on more than one thousand patients with thyroid nodules who received conventional open thyroidectomy, and found that about 28% of the patients had thyroid function damage after surgery, and required thyroxine, mainly due to the damage of parathyroid gland during surgery.^19^

In this study, patients who underwent US-guided RFA did not show significant changes in postoperative thyroid function, while patients who received conventional open thyroidectomy showed a significant decrease in thyroid function. Consistent with the study by Baldwin *et al*,^20^ our results confirm the ability of US-guided RFA to further reduce thyroid function damage. US-guided RFA implements precise puncture through ultrasound guidance. During the ablation process, when the needle tip reaches a certain temperature, the process will automatically stop without causing damage to normal thyroid tissue. Additionally, a liquid isolation zone that is established by injecting saline into the thyroid capsule further prevents any damage that may potentially affect thyroid function.^21^,^22^

Our results showed that both groups of patients had no evidence of recurrence 12 months after the operation, which is consistent with the study by Guo *et al*.^23^ Our results suggest that US-guided RFA can achieve similar surgical effects as conventional open thyroidectomy for BTN removal, and does not increase the rate of node recurrence. Taken together, our results confirm the efficiency of US-guided RFA, and suggest that this operation can be actively selected to further improve the clinical benefits of patients. It can provide a certain reference for clinical workers when undergoing thyroid surgery treatment.

### Limitations:

This is a single-center retrospective study with a small sample size and short (only 12 months) follow-up after surgery, and only a few indicators were studied, which may affect the objectivity and extrapolation of our conclusions.

## CONCLUSION

Compared with conventional OT, US-guided RFA has the advantages of less trauma, faster postoperative recovery, fewer complications, and less impact on thyroid function in the treatment of patients with BTN.

### Authors’ contributions:

**YF:** Conceived and designed the study.

**YX, HW and GZ:** Collected the data and performed the analysis.

**YF:** Was involved in the writing of the manuscript and is responsible for the integrity of the study.

All authors have read and approved the final manuscript.
